# Activation of Human Neutrophils by the Anti-Inflammatory Mediator *Esenbeckia leiocarpa* Leads to Atypical Apoptosis

**DOI:** 10.1155/2012/198382

**Published:** 2012-05-08

**Authors:** Rafael de Liz, Heros Horst, Moacir Geraldo Pizzolatti, Tânia Silvia Fröde, Denis Girard

**Affiliations:** ^1^Laboratoire de Recherche en Inflammation et Physiologie des Granulocytes, INRS-Institut Armand-Frappier, Université du Québec, 531 Boulevord des Prairies, Laval, QC, Canada H7V 1B7; ^2^Department of Clinical Analysis, Center of Health Sciences, Federal University of Santa Catarina, Campus Universitário, Trindade, 88040-970 Florianópolis, SC, Brazil; ^3^Department of Chemistry, Center of Physical and Mathematical Sciences, Federal University of Santa Catarina, Campus Universitário, Trindade, 88040-970 Florianópolis, SC, Brazil

## Abstract

Despite the fact that *Esenbeckia leiocarpa*, a Brazilian plant, possesses potential anti-inflammatory properties, its effect in neutrophils, key players in inflammation, has never been investigated. In this study, a crude hydroalcoholic extract (CHE) was used to evaluate the potential toxic or agonistic effect of *E. leiocarpa* in human neutrophils. At a noncytotoxic concentration of 500 **μ**g/mL, CHE increased actin polymerization and cell signaling events, especially p38 MAPK. Its modulatory activity on neutrophil cell apoptosis was investigated by cytology and by flow cytometry and, although CHE increased the apoptotic rate (by cytology) and increased annexin-V binding, it did not, unexpectedly, increase CD16 shedding. CHE increased the degradation of the cytoskeletal proteins gelsolin and paxillin but, surprisingly, not of vimentin. The proapoptotic activity of CHE was reversed by a pan-caspase inhibitor but not by a p38 inhibitor. We conclude that CHE is a novel human neutrophil agonist that induces apoptosis by a caspase-dependent and p38-independent mechanism in an atypical fashion based on its lack of effect on CD16 shedding and vimentin degradation. Since the resolution of inflammation occurs by elimination of apoptotic neutrophils, the ability of CHE to induce neutrophil apoptosis correlates well with its anti-inflammatory properties, as previously reported.

## 1. Introduction

Brazil boasts the greatest plant diversity on the world (approximately 55,000 species of higher plants) [1]. Brazilian plants represent a large source of potentially therapeutic compounds, a variety of which have been used in attempts to treat several infectious and/or inflammatory conditions. *Esenbeckia *species, such as *Esenbeckia leiocarpa* (Rutaceae), are wide spread in several Brazilian regions. The presence of various chemical constituents, primarily alkaloids, has already been demonstrated in this genus [2, 3]. In addition, coumarins [4], chalcones [4], as well as triterpenes and lignans have been shown to be, at least in part, responsible for different biological properties already attributed to this herb, including antimalarial [5], antiparasitic [6], antimicrobial [7], and anticholinesterasic [2] effects.

There are few studies investigating the anti-inflammatory effect of *Esenbeckia leiocarpa*. However, previous reports have singled out alkaloids as playing a pivotal role in inflammatory conditions. For instance, Jiao et al. [8] showed that some alkaloids promote a potent inhibitory activity on the production of nitric oxide (NO), tumor necrosis factor *α* (TNF-*α*), or interleukin 6 (IL-6), in mouse macrophage RAW264.7 cells stimulated by lipopolysaccharide (LPS) [8]. Others have demonstrated that the alkaloid 6-ethoxybuphanidrine was effective in inducing apoptosis in HL-60 and MDA-MB-231 tumor cell lines [9]. Other chemical constituents have caused antiproliferative and pro-apoptotic effects. Coumarins induced apoptosis in HeLa cells through a ROS-mediated mitochondrial dysfunction pathway. Moreover, chalcones were effective in inducing early apoptosis in HL60 cells [10, 11]. Recently, *Esenbeckia leiocarpa* was found to possess interesting anti-inflammatory activity *in vivo* since it protected against carrageenan-induced inflammation [12].

Polymorphonuclear neutrophil cells (PMNs) are known for their major role in inflammation. These cells are the first to arrive at inflammatory sites, where they secrete different cytokines/chemokines that attract either other PMNs or other leukocytes [13–15]. Because the resolution of inflammation is known to occur by the elimination of apoptotic PMNs by professional phagocytes, it is important to identify new agents that can induce or accelerate PMN apoptosis, since such agents could represent future potential therapeutic candidates [14–18]. In order to investigate the potential anti-inflammatory properties of *Esenbeckia leiocarpa *and to better understand the mode of action involved, we decided to determine whether or not a crude extract preparation of this plant could activate human PMNs and if it can accelerate their ability to undergo spontaneous apoptosis (SA). We found that the crude hydroalcoholic extract (CHE) of *Esenbeckia leiocarpa* bark can activate human PMNs by inducing actin polymerization, cell signaling events, and cleavage of some cytoskeletal proteins. Also, we demonstrate that CHE accelerates SA by a mechanism involving caspases but not p38 activation but also by a mechanism that does not increase vimentin cleavage and CD16 shedding.

## 2. Materials and Methods

### 2.1. Plant Materials

Samples of bark from *Esenbeckia leiocarpa* were collected in Arenápolis, a town located in the state of Mato Grosso, Brazil, collected in August 2007 and were identified by a biologist, Professor Dr. Celice Alexandre, of the State University of Mato Grosso, Tangará da Serra, MT, Brazil, where a voucher specimen (38639) was deposited. *Esenbeckia leiocarpa *barks were air-dried and protected from light at room temperature (25°C) for one week. In addition, the dried barks (5400 g) were ground into particles (1.5 mm) using a knife mill (Mill TE-651, Tecnal, Piracicaba, SP, Brazil). The ground material was extracted with 8 L of 96% ethanol (plant material: ethanol 1 : 8, w/v) at room temperature. After ten days, the extract obtained was filtered (using Whatman paper no. 1), and the ethanol was removed by rotavapor (Fisatom-802, São Paulo, SP, Brazil) at 55°C under reduced pressure (460 mmHg; Vacuum Q-355A2, Quimis, Diadema, SP, Brazil). In order to obtain a final lyophilized powder, this procedure was repeated three times in a period of one month, with a resulting yield of 290 g to the crude hydroalcoholic extract (CHE). CHE was dissolved in the diluent composed of HBSS-1% dimethyl sulfoxide (DMSO) (Sigma Chemical Company (St. Louis, MO, USA)).

### 2.2. Chemical and Agonists

The plant lectin *Viscum album* agglutinin 1 (VAA-I) used as an inducer of PMN apoptosis [19], dimethyl sulfoxide (DMSO), SB203580, a specific cell-permeable inhibitor of the MAP kinase homologues p38alpha, p38beta, and p38beta2, and PD98059, an inhibitor of MEK1 and MEK2, two enzymes leading to phosphorylation of ERK-1/2 and N-formyl-methionyl-leucyl-phenylalanine (fMLP), were purchased from Sigma Chemical Company (St. Louis, MO, USA). The FITC-phalloidin conjugate was purchased from Molecular Probes (Eugene, OR, USA). FITC-Annexin-V was purchased from BioSource International (Camarillo, CA, USA) and FITC-mouse anti-human CD16 mAb was purchased from BD Pharmingen (Mississauga, Ontario, Canada). Granulocyte macrophage colony-stimulating factor (GM-CSF), a classical PMN agonist and antiapoptotic agent, was purchased from PeproTech Inc (Rocky Hill, NJ, USA). The caspase-1, -3, -4, and -7 inhibitor N-benzyloxycarbonyl-V-A-D-O-methyl-fluoromethyl ketone (z-VAD-FMK) was purchased from Calbiochem (La Jolla, CA). The caspase-3 inhibitor z-Asp(OMe)-Gln-Met-Asp(OMe)-FMK (z-DQMD-FMK), the irreversible caspase-6 inhibitor z-Val-Glu(OMe)-Ile-Asp(OMe)-FMK (z-VEID-FMK), and the irreversible caspase-9 inhibitor z-Leu-Glu(OMe)-His-Asp(OMe)-FMK (z-LEHD-FMK) were purchased from Calbiochem (La Jolla, CA). The following mAbs to human cytoskeletal proteins were purchased from Sigma-Aldrich (St. Louis, MO): anti-gelsolin (clone GS-2C4), anti-paxillin (clone PXC-10), and anti-vimentin (clone V9).

### 2.3. Neutrophil Isolation

Cells were isolated from venous blood of healthy volunteers by dextran sedimentation followed by centrifugation over Ficoll-Hypaque (Amersham Pharmacia Biotech Inc., Baie d'Urfé, Québec, Canada), as described previously [20]. Blood donations were obtained from informed and consenting individuals according to our institutionally approved procedures. Cell viability (>98%) was monitored by Trypan blue exclusion, and the purity (>98%) was verified by cytology from cytocentrifuged preparations stained using the Hema-3 stain set (Biochemical Sciences Inc., Swedesboro, NJ, USA), according to the manufacturer's protocol.

### 2.4. Actin Polymerization

Freshly isolated human neutrophils (10 × 10^6^ cells/mL suspended in RPMI-1640) were incubated for short periods of time (5, 15 or 30 min.) at 37°C with buffer (DMSO 1%) or CHE (500 *μ*g/mL) in a final volume of 100 *μ*L. Synthetic peptide N-formyl-methionyl-leucyl-phenylalanine (fMLP) (a classical neutrophil activator) at the dose of 10^-8 ^M was used as positive control. After incubation of PMNs with buffer or CHE, digitonin and paraformaldehyde (PFA) were used for permeabilization and cell fixation, respectively. Cells were washed and incubated with phalloidin-FITC (binds to filamentous of actin) for 20 min at 4°C (light protected) prior to FACS analysis. Flow cytometric analysis (10,000 events) was performed using a FACScan (BD Biosciences, Sao Jose, CA, USA).

### 2.5. Tyrosine Phosphorylation Events

Neutrophils (10 × 10^6^ cells/mL in RPMI-1640) were incubated for 0.5, 1, 5, 15, 30, 45, or 60 min at 37°C with the diluent (HBSS-1%DMSO), GM-CSF (65 ng/mL), or CHE (500 *μ*g/mL) in a final volume of 100 *μ*L. Reactions were stopped by adding 35 *μ*L of a 4X Laemmli sample buffer. Aliquots corresponding to 1 × 10^6^ cells were loaded onto 10% SDS-PAGE and transferred from gel to nitrocellulose membranes (Amersham Pharmacia Bio-tech Inc., Baie d'Urfé, Québec, Canada). Nonspecific sites were blocked with 3% bovine serum albumin (BSA) in TBS-Tween (25 mM Tris-HCl, pH 7.8, 190 mM NaCl, 0.15% Tween-20) for 1 h at room temperature. The monoclonal antiphosphotyrosine (clone 4G10 (1 : 1000)) was then incubated with membranes for 1 h at 37°C followed by washes and incubated with a horseradish peroxidase-labelled goat anti-mouse IgG (1 : 15,000, Bio/Can) for 1 h at room temperature in fresh blocking solution. Membranes were washed three times with TBS-Tween, and phosphotyrosine bands were revealed with the enhanced chemiluminescence (ECL) Western blotting detection system (Amersham Pharmacia Biotech Baie d'Urfé, Québec, Canada). Protein loading was verified by staining the membranes with Coomassie blue at the end of the experiments. 

### 2.6. Activation of p38 and ERK-1/2

Neutrophils (10 × 10^6^ cells/mL) were isolated and incubated as above at 37°C with buffer (DMSO 1%) or CHE (500 *μ*g/mL) in a final volume of 100 *μ*L. Reactions were stopped by adding 35 *μ*L 4X Laemmeli's sample buffer, as described previously [21]. Samples corresponding to 1 × 10^6^ cells were loaded onto 10% SDS-PAGE and transferred from gel to nitrocellulose membranes (Amersham Pharmacia Bio-tech Inc.). Nonspecific sites were blocked with 3% bovine serum albumin (BSA) in TBS-Tween (25 mM Tris-HCl, pH 7.8, 190 mM NaCl, 0.15% Tween-20) and Western blots were performed as described previously by Pelletier et al., in 2002 [21]. Monoclonal antiphospho-p38 (pTpY^180/182^) antibody (1 : 1000; Biosource, Camarillo, CA, USA) and HRP-conjugated goat anti-mouse (1 : 15,000), or monoclonal anti-phosphospecific ERK-1/2 MAPK (clone 12D4) (Upstate cell signalling, Lake Placid, NY, USA) and HRP-goat anti-rabbit (1 : 15,000), both diluted in 3% nonfat dry milk, were used. Membranes were stripped for 30 min at 55°C with stripping buffer (100 mM 2-ME, 2% SDS, 62.5 mM Tris, pH 6.7), washed, and reprobed with an anti-p38 (clone C-20: sc-535) (1 : 1000; Santa Cruz Biotechnology, Santa Cruz, CA, USA), or polyclonal anti-ERK-1/2 (Millipore, Billerica, MA, USA) antibodies followed by an HRP-conjugated goat anti-mouse IgG + IgM (1 : 20,000; Jackson ImmunoResearch Laboratories, Inc.). p38 and Erk-1/2 proteins expression was revealed with ECL as per manufacturer's instructions.

### 2.7. Assessment of Neutrophils Apoptosis by Cytology and by Flow Cytometry

PMNs (100 *μ*L of a 10 × 10^6^ cells/mL suspension in RPMI-1640 supplemented with 10% autologous serum) were incubated with or without CHE (500 *μ*g/mL) for indicated times in the presence or absence of caspase-1, -3, -4, and -7 inhibitor, N-benzyloxycarbonyl-V-A-D-O-methyl-fluoromethyl ketone (z-VAD-FMK), caspase-3 inhibitor z-Asp(OMe)-Gln-Met-Asp(OMe)-FMK (z-DQMD-FMK), irreversible caspase-6 inhibitor z-Val-Glu(OMe)-Ile-Asp(OMe)-FMK (z-VEID-FMK), or irreversible caspase-9 inhibitor z-Leu-Glu(OMe)-His-Asp(OMe)-FMK (z-LEHD-FMK), or appropriate controls, as indicated in the figure legends, and apoptosis was evaluated by cytology and/or flow cytometry. Cytocentrifuge preparations of neutrophils were performed with a Cyto-tek centrifuge (Miles Scientific) essentially as previously described and were stained with a Diff-Quick staining kit (Baxter Healthcare Corporation, Miami, FL, USA), according to the manufacturer's instructions. Cells were examined by light microscopy at 400x final magnification, and apoptotic neutrophils were defined as cells containing one or more characteristic darkly stained pyknotic nuclei. An ocular containing a 10 × 10-square grill was used to count at least five different fields (>100 cells) for assessment of apoptotic cells. Results were expressed as percentage of apoptotic cells. For the flow cytometric procedure, apoptosis was investigated using fluorescence labelling with Annexin-V-FITC binding or anti-CD16-FITC as previously published [22]. Ten thousand cells were analyzed by FACS Calibur (Becton-Dickinson, San Jose, CA) using CellQuest program (BD Biosciences, Sao Jose, CA, USA). The number of late apoptotic and necrotic cells was subtracted in order to evaluate the percentage of apoptotic cells.

### 2.8. Degradation of Cytoskeletal Proteins

Neutrophils (10 × 10^6^ cells/mL in a 96-well plate) were incubated with or without 1000 ng/mL VAA-I or 500 *μ*g/mL CHE in its diluent (1% DMSO) for the indicated time period and then harvested for the preparation of cell lysates in Laemmli's sample buffer (Figure 5). Aliquots corresponding to 500,000 cells were loaded onto 10% SDS-PAGE and transferred from gel to nitrocellulose membranes (Amersham Pharmacia Bio-tech Inc, Baie d'Urfé, QC, Canada). Nonspecific sites were blocked with 3% BSA in TBS-T for 1 h at room temperature. Membranes were incubated with monoclonal anti-human cytoskeletal Abs (anti-gelsolin, anti-paxillin, anti-vimentin, and anti-vinculin) in the dilution of 1 : 1000 for 1 h at room temperature, followed by 3 washes, and incubated with an HRP-labelled goat anti-mouse IgG (1/15,000; BIO/CAN, Montreal, Canada) for 1 h at room temperature in fresh blocking solution [23]. Membranes were washed three times with TBS-T and bands were revealed with the ECL-Western blotting detection system (Pharmacia Biotech). Protein loading was verified by staining the membranes with Coomassie blue at the end of the experiments.

## 3. Results

### 3.1. CHE Is a Human Neutrophil Activator

**Figure 1:**
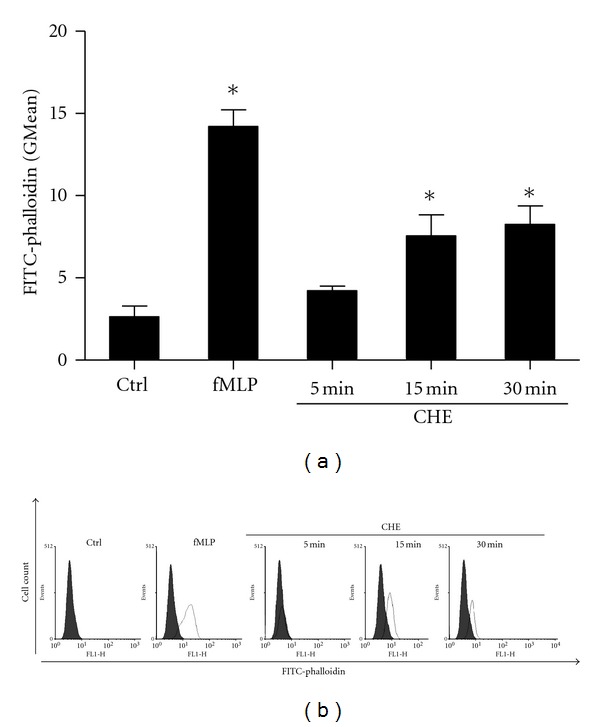
CHE induces actin filament polymerization in human PMNs. Freshly isolated human neutrophils were isolated and incubated (10 × 10^6^ cells/mL) with the diluent (HBSS-1% DMSO, Ctrl), crude hydroalcoholic extract (CHE) (500 *μ*g/mL), or fMLP (10^−8 ^M) for 5, 15, or 30 min. Actin polymerization was evaluated by monitoring FITC-phalloidin expression by flow cytometry. (a) Results are means ± SEM (*n* = 3). (b) Results shown are from one representative experiments plotted in (a). *P < 0.05 (versus Ctrl) by Student's *t*-test.

Because this was the first time that CHE was tested in human PMNs, we first determined its potential cytotoxicity. To do so, freshly isolated PMNs were incubated *in vitro* with increasing concentrations of CHE (0–1000 *μ*g/mL) over time (0–24 h) and necrosis was assessed by a trypan blue exclusion assay. Cell necrosis never exceeded 3% at concentrations ≤500 *μ*g/mL, and the number of cells initially seeded remained stable in plates observed 24 h later (*data not shown*). Cell necrosis never exceeded 5% when cells were incubated with CHE at concentrations greater than 500 *μ*g/mL for shorter periods of time (less than 60 min), but up to 15% of cells displayed necrosis at the highest concentration tested (1000 *μ*g/mL) after 24 h of incubation (*data not shown*). Based on these results, we selected the concentration of 500 *μ*g/mL for the remainder of this study, unless otherwise specified. Next, we determined whether or not CHE could induce actin polymerization, a typical assay for evaluating PMN activation [24, 25]. As illustrated in Figure 1, CHE increased actin polymerization, but this response required about 15 min to occur, as judged by the shift of fluorescence following phalloidin staining.

### 3.2. CHE Induces Phosphorylation Events: Activation of p38 but Not ERK-1/2 MAPKs

**Figure 2:**
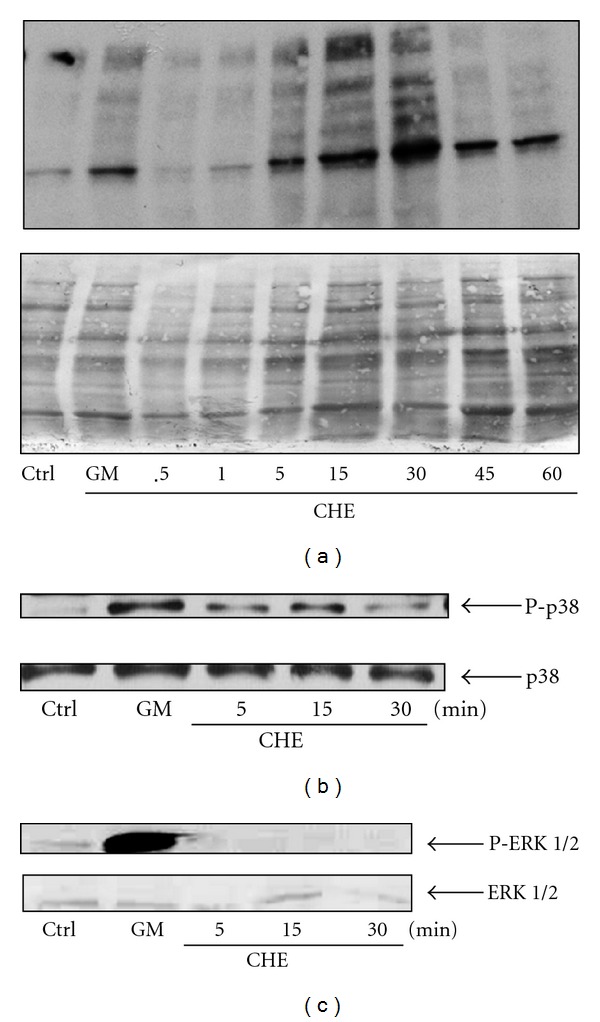
CHE induces tyrosine phosphorylation events in human neutrophils. (a), Freshly PMNs were isolated and incubated (10 × 10^6^ cells/mL) with the diluent (HBSS-1% DMSO, Ctrl), granulocyte macrophage colony-stimulating factor (GM) (65 ng/mL) (lane 2), and crude hydroalcoholic extract (CHE) (500 *μ*g/mL) at the periods of stimulation of 0.5, 1, 5, 15, 30, 45, or 60 min. Immunoblotting was performed as described in Section 2. Top panel: tyrosine phosphorylation of intracellular proteins; bottom panel, Coomassie blue staining of the membrane to indicate equivalence in loading. (b) and (c) cells were stimulated for 10 min with the diluent (Ctrl), GM-CSF (GM, 65 ng/mL), or with CHE (500 *μ*g/mL) for 5, 15 or 30 min. Immunoblotting was performed as described in Section 2. (b) and (c) Top panels illustrate the membranes that were revealed with antiphosphorylated p38 (b) or antiphosphorylated ERK-1/2 (c) antibodies; bottom panels are the corresponding membrane stained with the unphosphorylated form of p38 or ERK-1/2, respectively, indicating equivalent loading. Results shown are representative of at least three different experiments.

The aforementioned results strongly suggested that CHE could modulate PMN functions. We next evaluated the possibility that CHE activated phosphorylation events, since this cell signaling is important for modulating PMNs functions. As illustrated in Figure 2(a), CHE rapidly activated PMNs, as judged by increased signal intensity of total phosphotyrosine proteins detected by western blot after 30 seconds, peaking at 15–30 minutes and then declining after 45 min. Because p38 and ERK-1/2 MAPKs play significant roles in human PMN activity, we next determined whether or not CHE activated these kinases. CHE was found to activate p38 (Figure 2(b)) but not ERK-1/2 (Figure 2(c)), despite the fact that, as expected, the cytokine GM-CSF strongly induced phosphorylation of ERK-1/2. 

### 3.3. CHE: an Atypical Inducer of PMN Apoptosis

**Figure 3:**
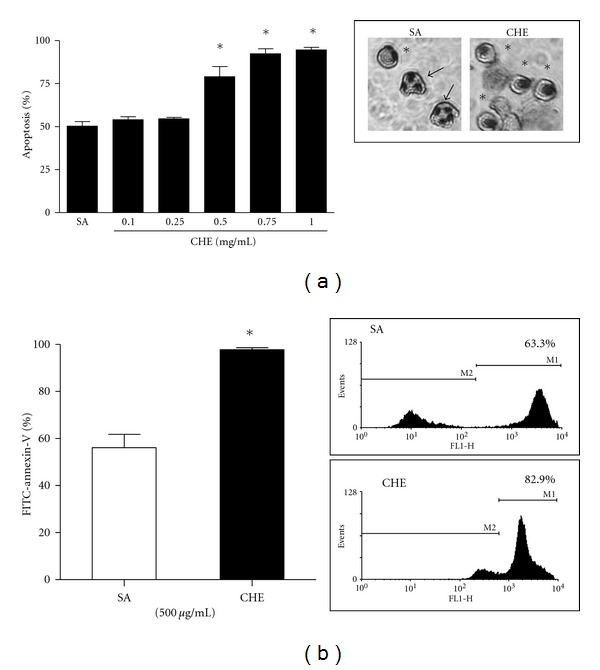
CHE induces apoptosis in human neutrophils. Freshly isolated human neutrophils were isolated and incubated (10 × 10^6^ cells/mL) with the diluent (SA) or with the indicated concentrations of CHE for 22 h. Apoptosis was evaluated by cytology (a) or by flow cytometry (b) using FITC-annexin-V as described Section 2. Inset in (a): picture of apoptotic (asterisks) or nonapoptotic (arrows) PMNs. Inset in (b): typical results obtained by FACS analysis from one experiments plotted in the corresponding bar graph. Results are means ± SEM (*n* ≥ 3). SA: spontaneous apoptosis (cells incubated with the diluent). *P < 0.05 versus SA by Student's *t*-test.

**Figure 4:**
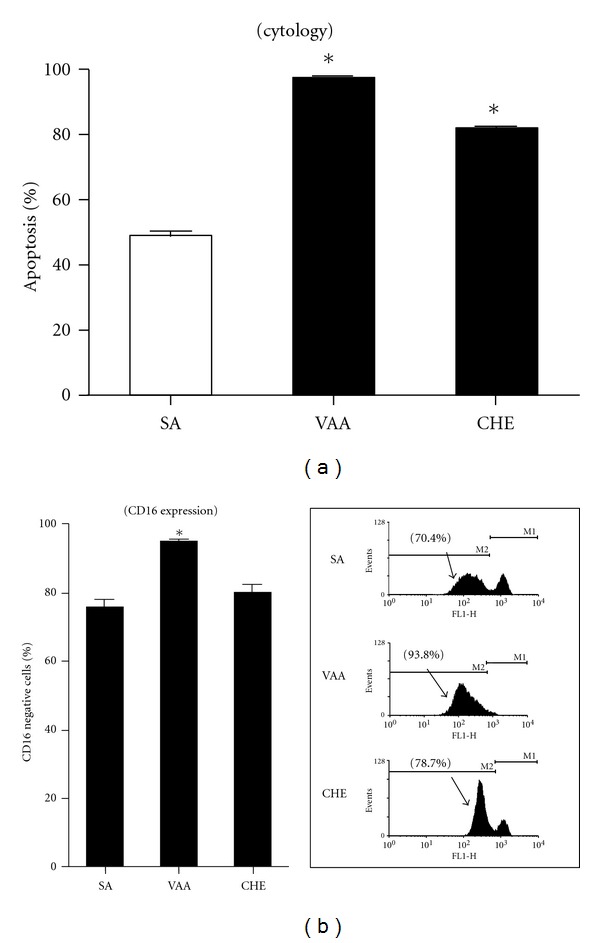
The pro-apoptotic activity of CHE does not correlate with CD16 shedding. Cells (10 × 10^6^ cells/mL) were incubated with the diluent (SA), 1000 ng/mL *Viscum album* agglutinin-I (VAA), or 500 *μ*g/mL CHE for 22 h and apoptosis was evaluated in parallel for each blood donors by cytology (a) or by monitoring CD16 cell surface expression by flow cytometry (b). Inset in (b): typical results obtained by FACS analysis and plotted in the corresponding bar graph. Results are means ± SEM (*n* = 4). *P < 0.05 versus SA by Student's *t*-test.

**Figure 5:**
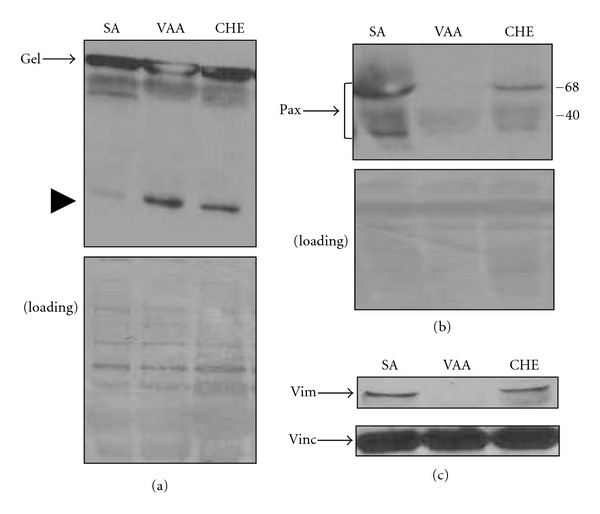
CHE increases the degradation of the cytoskeletal proteins gelsolin and paxillin but not vimentin during apoptosis. Cells were incubated (10 × 10^6^ cells/mL) with the diluent (DMSO (1%, SA), *Viscum album* agglutinin-I (VAA) (1000 ng/mL) or 500 *μ*g/mL CHE for 22 h. Degradation of gelsolin (Gel) (a), paxillin (Pax) (b), vimentin (Vim), or vinculin (Vinc) (c) was monitored by Western blot, as described in Section 2. Results are from one representative experiment of four. The position of the two paxillin fragments known to be recognized by the antibody (clone PXC-10) is illustrated. Bottom panels were stained with Coomassie blue at the end of the experiment in order to verify equivalent loading, except in (c), where the membranes were striped and probed with an antivinculin antibody for determining the equivalent loading. Note the appearance of the gelsolin fragment when cells were treated with VAA or CHE ((a), arrowhead).

Several assays were used to evaluate the effect of CHE on PMN apoptosis. As assessed by cytology, CHE significantly induced apoptosis at a concentration of ≥500 *μ*g/mL, where 79.0 ± 6.0% (mean ± SEM, *n* = 5) of cells were typically in apoptosis (pyknotic nuclei) versus 50.3 ± 2.6% for controls or SA (Figure 3(a)). We then evaluated apoptosis by flow cytometry with FITC-annexin-V, known to be increased at the apoptotic cell surface. As expected, a greater number of PMNs were annexin-V-positive when treated with 500 *μ*g/mL CHE versus SA (97.8% ± 0.9 versus 56.1% ± 5.6 for SA) (Figure 3(b)). In contrast to annexin-V, CD16 is known to be shed from the cell surface of apoptotic PMNs, leading to a decrease in expression [26]. Unexpectedly, we found that CD16 levels were not significantly decreased in CHE-induced PMNs (*data not shown*). We then conducted a series of experiments in which apoptosis was evaluated in parallel using cytology and flow cytometry, to monitoring CD16 cell surface expression in apoptotic cells. As expected, VAA-I, a potent pro-apoptotic plant lectin, induced apoptosis as assessed by cytology (Figure 4(a)) and decreased the cell surface expression of CD16 (Figure 4(b)) as compared to SA, whereas CHE induced apoptosis as determined by cytology, but levels of cell surface expression of CD16 did not significantly decrease in PMNs isolated from the same blood donors as compared to SA. Since these latter results suggested that CHE induced atypical PMN apoptosis, we then investigated the degradation of cytoskeletal proteins, an event characteristic of apoptosis. As shown in Figure 5, CHE induced the degradation of gelsolin as evidenced by the appearance of the 41 kDa fragment [19] and the degradation of the two main 60 and 48 kDa paxillin polypeptides recognized by the antibody [27]. These results were not surprising, since CHE is a pro-apoptotic molecule. Unexpectedly, CHE did not induce the degradation of the intermediate filament protein vimentin, despite the fact that, as expected, the pro-apoptotic VAA-I molecule induced its cleavage [27]. 

### 3.4. Involvement of Caspases in CHE-Induced PMN Apoptosis

**Figure 6:**
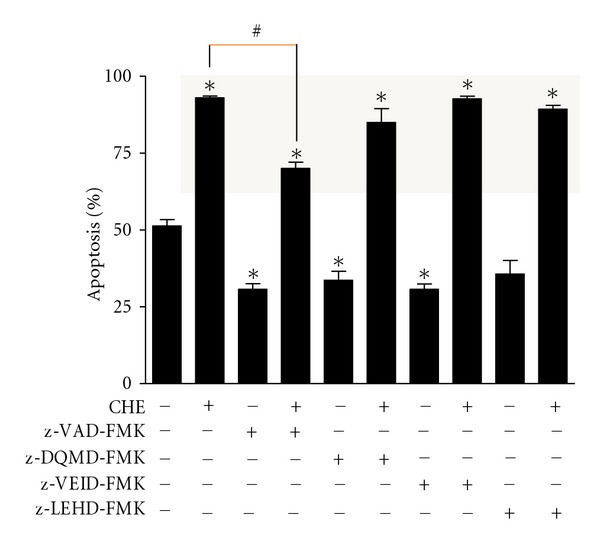
CHE induces apoptosis by a caspase-dependent mechanism. Cells (10 × 10^6^ cells/mL) were incubated with (+) or without (−) CHE in the presence (+) or absence (−) of a given caspase inhibitor: z-VAD-FMK (pan-caspase), z-DQMD-FMK (caspase-3), z-VEID-FMK (caspase-6), or z-LEHD-FMK (caspase-9) for 22 h. Apoptosis was evaluated by cytology. Results are means ± SEM (*n* = 4). *P < 0.05 versus Ctrl (left column, absence of CHE and inhibitors) and ^#^ *P* < 0.05 versus CHE by Student's *t*-test.

Since CHE induced the cleavage of gelsolin and paxillin, an event requiring caspase activation [19, 27], we next decided to investigate whether or not CHE acted via caspases. CHE-induced PMNs were incubated in the presence or absence of a pan-caspase inhibitor (z-VAD-fmk) or a caspase-3 (z-DQMD-FMK), caspase-6 (z-VEID-FMK), or caspase-9 (z-LEHD-FMK)-specific inhibitor and apoptosis was evaluated by cytology. As expected, the tested inhibitors suppressed SA, but only the pan caspase inhibitor was able to reverse the pro-apoptotic activity of CHE when the apoptotic rate was 70% rather than close to 90% (grey zone in Figure 6). All other inhibitors did not significantly reverse the ability of CHE to induce apoptosis.

### 3.5. CHE Induced Apoptosis by a p38-Independent Mechanism

**Figure 7:**
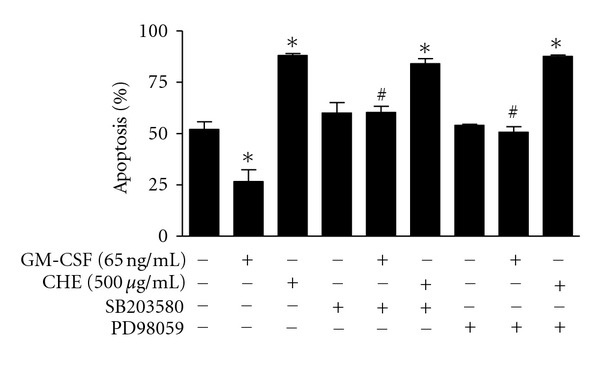
CHE induces apoptosis in a p38-independent mechanism. Cells (10 × 10^6^ cells/mL) were incubated with (+) or without (−) CHE or the antiapoptotic cytokine GM-CSF (GM, 65 ng/mL) in the presence (+) or absence (−) of the p38 inhibitor (SB203580) or the MEK-1/MEK-2 inhibitor (PD98059) for 22 h. Apoptosis was evaluated by cytology. Results are means ± SEM (*n* = 4). *P < 0.05 versus Ctrl (left column, absence of CHE and inhibitors) and ^#^ *P* < 0.05 versus GM by Student's *t*-test.

We then decided to determine whether or not p38 was involved in CHE-induced neutrophil apoptosis, since this kinase is activated by CHE. However, as illustrated in Figure 7, treatment with the p38-specific inhibitor (SB203580) did not reverse the pro-apoptotic activity of CHE, despite the fact that this inhibitor, as well as the MEK-1/MEK-2 inhibitor, was able to reverse the antiapoptotic activity of the cytokine GM-CSF.

## 4. Discussion

During apoptosis, PMNs are known to shrink and cytoskeleton breakdown occurs, as has been shown by the cleavage of several cytoskeletal proteins largely associated with caspase activity [19, 27–29]. Because of the importance of eliminating apoptotic PMNs by professional phagocytes for the resolution of inflammation [13, 14, 16, 18, 30], it is critical to identify new agents that may act as neutrophil activators and, more specifically, that may act as regulators of PMN apoptosis, and to understand their modes of action.

This is the first study to investigate the role of the CHE from *Esenbeckia leiocarpa* bark in human neutrophil cell physiology, an extract that possesses interesting anti-inflammatory properties [31]. Clearly, CHE is a novel PMN activator, since it induced actin polymerization and cell signaling events, including p38 activation. Furthermore, CHE induced cleavage of cytoskeletal proteins and PMN apoptosis. The fact that the pro-apoptotic activity of CHE was reversed by the z-VAD-FMK pan-caspase inhibitor (known to inhibit the effector caspase-1, -3, -4, and -7) suggests that several caspases could be simultaneously or sequentially activated and also that several pathways of cell apoptosis may be involved. Caspase-3 is known to be a central player in both classical extrinsic and intrinsic pathways of cell apoptosis [32]. In addition, caspase-4 activation is known to be a major event occurring during the more recently identified pathway of cell apoptosis: the endoplasmic reticulum (ER) stress-induced apoptosis. Only recently have there been some evidences of the ER stress-induced pathway in human PMNs [33]. In contrast to z-VAD-FMK, the more specific inhibitors used in this study (those to caspase-3, -6, or -9) were not able to reverse the CHE pro-apoptotic activity. Because CHE accelerated SA, and the three main pathways of cell apoptosis were activated during SA, it is plausible that inhibiting only one caspase with a more specific inhibitor than the pancaspase was not sufficient to reverse the effect of CHE. In addition, CHE is a crude extract composed of several hundred chemical compounds; because of this, it is plausible that some compounds preferentially activate a given cell apoptotic pathway, while others activate several pathways leading to the inability of a particular caspase inhibitor to reverse the pro-apoptotic activity of CHE. However, the fact that treatment with the p38 inhibitor (SB203580) did not reverse the pro-apoptotic activity of CHE led us to conclude that CHE induced PMN apoptosis by a p38-independent mechanism, and that p38 is probably involved in other PMN functions, although this remains to be determined. Several MAPKs, including p38 and ERK-1/2, are known to be involved in the regulation of PMN apoptosis [32]. ERK-1/2 activation is normally implicated in PMN survival, while activation of p38 is observed in PMNs in response to treatment with pro- or antiapoptotic agents. Thus, the role of p38 in the regulation of PMN apoptosis is unclear and the present data support further our hypothesis that p38 is involved in functions other than apoptosis in human PMNs.

The mode of action of CHE for inducing apoptosis is not a conventional one. In general, pro-apoptotic agents increase CD16 shedding in human PMNs [26], but CHE did not alter CD16 cell surface expression when compared to SA. However, CHE increased cell surface expression of annexin-V and induced typical morphological changes, as evidenced by the appearance of pyknotic nuclei. Although the mechanism involved in CD16 shedding is not fully defined, it appears that a metalloproteinase is responsible for CD16 shedding [34, 35]. Of note, it has been reported previously that CD16 shedding is not only inhibited by metalloproteinase inhibitors, but also by serine protease inhibitors indicating inhibited that this phenomenon is very complex [35, 36]. Furthermore, a link between cytoskeleton rearrangement and the induction of CD16 shedding has been proposed, and actin polymerization has been found to induce shedding of CD16 [24]. This was determined with the use of jasplakinolide, an actin-polymerizing peptide, where enhanced actin polymerization was found to induce time- and concentration-dependent shedding of CD16. Therefore, the fact that CHE did not increase CD16 shedding but induced actin polymerization is another criterion indicating that CHE induces atypical apoptosis in PMNs. In addition, although CHE induced the degradation of the cytoskeletal proteins gelsolin and paxillin, as expected for pro-apoptotic molecules, it did not induce the degradation of vimentin that is normally increased by pro-apoptotic agents when compared to SA [23, 27].

In conclusion, the results of this study clearly establish that CHE is a novel PMN activator. Although its mode of action has not yet been fully defined, we demonstrated that CHE induced tyrosine phosphorylation events, including p38 activation, and targeted the cytoskeleton, as evidenced by actin polymerization and degradation of cytoskeletal proteins. CHE induces apoptosis in PMNs by a caspase-dependent and p38-independent mechanism but acted as an atypical pro-apoptotic agent, based on its inability to increase CD16 shedding and vimentin degradation. It is not without any precedent that a proapoptotic agent induces apoptotis in human PMNs, since we have previously demonstrated that arsenic trioxide also activates p38, but not ERK [37]. We conclude that CHE possesses *in vitro* anti-inflammatory properties, based on its ability to induce PMN apoptosis, and this may be, at least in part, responsible for its previously reported *in vivo* anti-inflammatory activity.
